# Intratumoral delivery of PD-1/PD-L1 and CTLA-4 inhibitors for recurrent/refractory solid tumors: a proof-of-concept treatment strategy

**DOI:** 10.3389/fimmu.2025.1669924

**Published:** 2025-12-12

**Authors:** Hongye Tan, Noor Ul Huda Shah, Bingjia He, Tianrun Liu, Tianheng Li, Manting Liu, Yingying Gu, Cheng Zhi, Yongqiong Ou, Junhao Huang, Ming Li, Shenghua Zuo, Dongni Chen, Ruzhai Qin, Hainan Yang, Xufeng Li, Hui Lian, Qingde Wu, Rom Leidner, Rui Chen, Ankui Yang, Lili Yang, Zhenfeng Zhang

**Affiliations:** 1 Department of Radiology, Translational Medicine Center, Guangzhou Key Laboratory for Research and Development of Nano-Biomedical Technology for Diagnosis and Therapy, Guangdong Provincial Education Department Key Laboratory of Nano-Immunoregulation Tumor Microenvironment, Central Laboratory, the Second Affiliated Hospital of Guangzhou Medical University, Guangzhou, China; 2 Department of Thyroid Surgery, Sun Yat-Sen Memorial Hospital, Sun Yat-sen University, Guangzhou, China; 3 Guangdong Provincial Key Laboratory of Food, Nutrition and Health, Department of Nutrition, School of Public Health, Sun Yat-sen University, Guangzhou, China; 4 Department of Pathology, the Second Affiliated Hospital of Guangzhou Medical University, Guangzhou, China; 5 Center for Clinical Trial, the Second Affiliated Hospital of Guangzhou Medical University, Guangzhou, China; 6 Department of Radiology, Shunde Chinese Medicine Hospital, the Affiliated Hospital of Traditional Chinese Medicine University of Guangzhou, Foshan, China; 7 Earle A. Chiles Research Institute, Providence Cancer Institute, Portland, OR, United States; 8 Department of Respiratory and Critical Care Medicine, Sun Yat-Sen Memorial Hospital, Sun Yat-Sen University, Guangzhou, China; 9 Department of Head and Neck Surgery, State Key Laboratory of Oncology in South China; Collaborative Innovation Center for Cancer Medicine, Sun Yat-Sen University Cancer Center, Guangzhou, China

**Keywords:** PD-1/PD-L1 inhibitors, CTLA-4 inhibitor, recurrent/refractory advanced solid tumor, intratumoral injection, clinical trial

## Abstract

**Background:**

Immune checkpoint inhibitors (ICIs) are a leading immunotherapy. However, their application has not universally translated into significant benefits. A substantial number of patients either show resistance or relapse post-initial response, which emphasizes the need for more sophisticated therapeutic approaches. The drug combination is one promising route. The cancer-immunity cycle reveals the anti-tumor immune-related rate limiting steps of tumors. Theoretically, focusing on different phases of the cancer-immunity cycle can enhance therapeutic results. However, combination therapy includes a higher risk of adverse effects, which demand careful consideration.

**Methods:**

In this study, we combined programmed death-1 (PD-1)/ programmed death ligand-1 (PD-L1) and cytotoxic T Lymphocyte antigen 4 (CTLA-4) inhibitors with a reduced dosage but via an intra-tumor drug delivery strategy to treat recurrent/refractory (R/R) advanced solid tumors.

**Results:**

Herein, we report four patients with favorable outcomes (complete response for more than 2 years). In our cases, most TRAEs are of grade 1-2.

**Conclusion:**

Intratumoral co-delivery of PD-1/PD-L1 and CTLA-4 inhibitors with reduced dosage shows promising efficacy and safety in R/R advanced solid tumors. In addition to reducing drug-related adverse events, this technology has advantages in activating tumor immunity.

**Clinical Trial Registration:**

https://clinicaltrials.gov/, identifier: NCT03755739.

## Introduction

Chemotherapy or radiotherapy is a main option for patients with R/R solid tumors, who usually have a poor prognosis with a median overall survival (mOS) period of 10–12 months (1). The application of immune checkpoint inhibitors (ICIs) improved tumor outcomes. Compared with chemotherapy alone, adding immunotherapy to the initial treatment of advanced and metastatic malignant tumors such as endometrial cancer can prolong the overall survival (OS) (2). However, the majority of patients responded poorly to immunotherapy, falling short of expected results (3).

CTLA-4 is believed to regulate T cell maturation and proliferation in the early stages of immune response (mainly in lymph nodes) and PD-1 inhibits T cells function in the later stages of immune response (mainly in peripheral tissues) (4). A clinical trial demonstrated that the combination of nivolumab (a PD1 inhibitor) plus ipilimumab (a CTLA4 inhibitor) increased the 5-year survival rate compared to chemotherapy for metastatic non-small cell lung cancer (mNSCLC) (5). This combination also improved median event-free survival (EFS) and pathologic complete response rates of resectable NSCLC (6). Another study suggests that the combination of CTLA-4 and PD-1 blockade can reverse the primary resistance to PD-1 blockade therapy in some patients (7). The combination of CTLA-4 and PD-1 inhibitors may induce greater T cell proliferation and reduce regulatory T (T_reg_)-mediated immunosuppression. This complementary mechanism is capable of improving clinical outcomes in patients with diverse types of cancer (8).

Although the double-ICI combinations have exhibited promising therapeutic efficacy, they are accompanied by a higher incidence of toxicity compared with single-agent immunotherapy (9, 10). For CTLA-4 inhibitors via intravenous route, 60-65% of patients experienced widespread immune-related adverse events (irAEs) in previous clinical studies (11). The combination of CTLA-4 and PD-1 inhibitors achieved a higher risk of irAEs of 95.5% (12). Such combination also leads to an increased incidence of severe adverse events (SAE) and a higher rate of drug discontinuation (13). Combination therapy requires a safer route of administration. Directly injecting drugs into tumors can obtain high concentrations of immunostimulatory products *in situ* by using a small dosage of drugs, while also compensating for the serious systemic exposure and off-target toxicity caused by intravenous administration (11).

In this study, we design a proof-of-concept treatment strategy: intratumor injection guided by CT scanning for delivery of PD-1 or PD-L1 inhibitor plus CTLA-4 inhibitor to initiate or reinitiate a better and safer anti-tumor immune system. Among patients with advanced solid tumors in our center treated with this strategy, some patients achieved better results. We reported the application of this combination in four patients who all achieved complete response (CR) with longer survival.

## Case report

**Figure 1:**
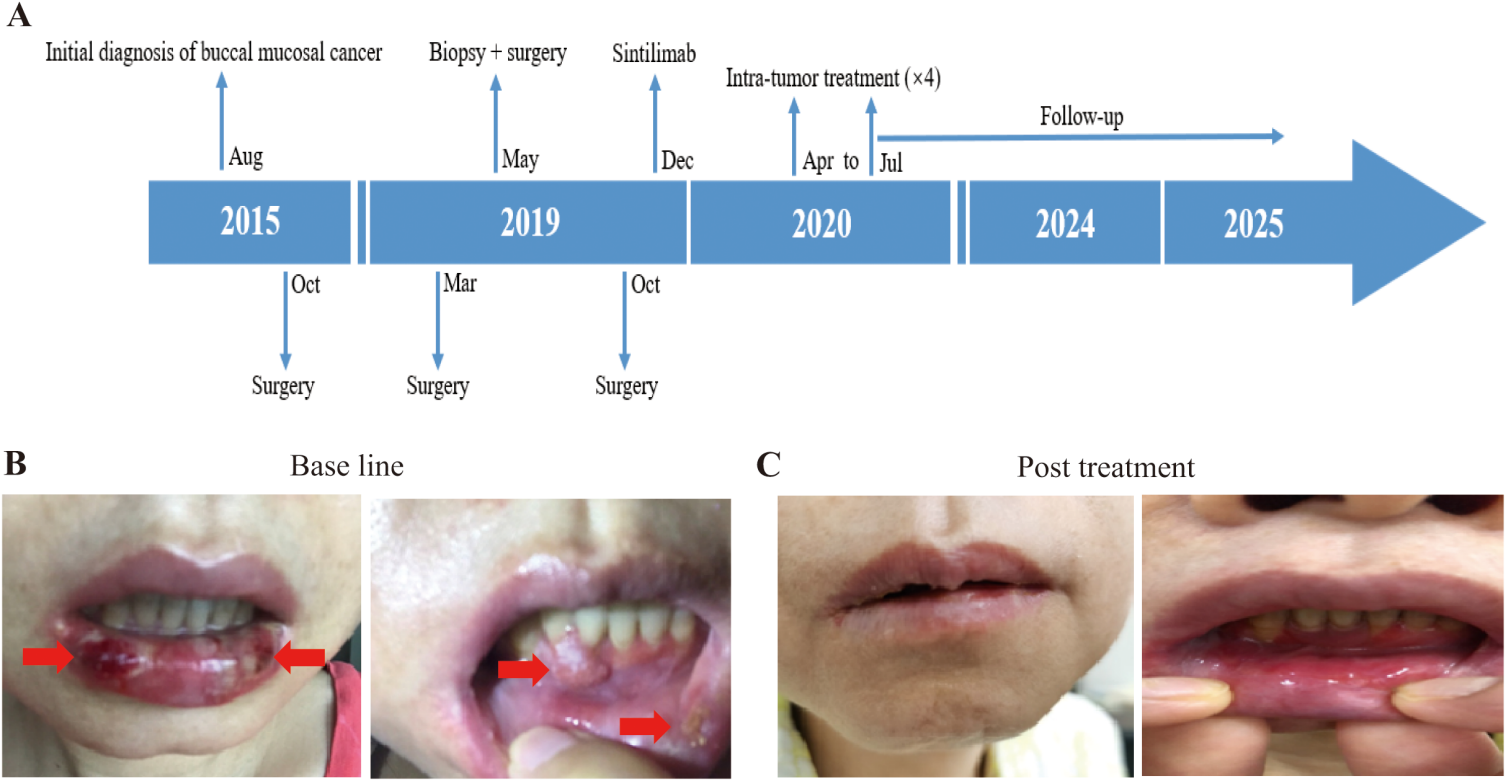
Illustration of the treatment process of combination therapy for Patient 1. **(A)** The treatment timeline for Patient 1 is outlined, showing the progression from initial conditions to post-treatment outcomes. **(B)** Prior to treatment, the patient exhibited multiple white patches in the mouth, along with a noticeable lump in the lower gums. The white patches and lump in the lower gums is indicated by the red arrow. **(C)** Following treatment with combination of 4ml pembrolizumab and 5ml ipilimumab, both the mucosal leukoplakia and gingival mass disappeared in the patient.

Patient 1 was a 42-year-old female who was diagnosed with buccal mucosal squamous cell carcinoma (SCC) in August 2015. She initially found a firm ulcerated mass 2x1cm in size in the posterior left buccal mucosa, 2 leukoplakia lesions about 0.5 cm in sizes in the left buccal mucosa 5 cm away from the ulcerated mass and a 1 cm leukoplakia of the right buccal mucosa. Biopsy pathology in September 2015 showed carcinoma *in situ* on the 2 leukoplakia lesions of the left buccal mucosa and then surgery was performed in October 2015 with wide local excision of all the lesions on both buccal mucosae. All margins of excisions were negative and no lymph nodes were dissected in the surgery. Post resection pathological staging was squamous cell carcinoma *in situ* (TisN0M0) on the left buccal mucosa and the leukoplakia lesion on right buccal mucosa was precancerous dysplasia with chronic inflammation. The patient had a recurrent SCC of the right buccal mucosa in March 2019, which prompted second surgery with wide local excision without local lymph node dissection. Its pathology indicated SCC *in situ* (TisN0M0) with partly heavy dysplasia and partial margin presented mild-heavy dysplasia. However, in May 2019, right cheek nodules adjacent to the excision site tested positive for recurrent SCC on biopsy at an USA hospital; a lower gingiva lesion was suspected but not confirmed on enhanced MRI. The patient had undergone oral cavity composite lesion resection (including right buccal mucosa and marginal mandibulectomy), right neck lymph nodes dissection (levels 1-3), dental extractions (teeth #2, 3, 30, 31), and biopsy of a raised lesion in lower anterior gingiva midline at the USA hospital. The pathology reported SCC of a firm ulcerated mass in the posterior right buccal mucosa, raised lesions of right oral commissure and lower anterior gingiva midline, and a leukoplakia of the left buccal mucosa, with pathological staging as pT3(m)N0M0. In October 2019, she returned Shanghai and underwent surgical removal of the lower anterior gingival mass. Radiotherapy and chemotherapy would be the next steps, but the patient refused due to the limited efficacy and irreversible damage to oral function considered by several oncologists consulted. In December 2019, based on positive PD-L1 expression (TPS>50%), she initiated intravenous sintilizumab (a PD-1 monoclonal antibody, 200mg every 21–29 days) (**Figure 1A**). During 6 times immunotherapy, the tumor marker CA242 did not show significant changes (from 37 to 43 u/ml). But she developed grade 3 immune-related mucosal inflammation and enteritis per CTCAE v5.0 criteria, leading to treatment discontinuation in April 2020. In order to continue receiving immunotherapy, she participated in our trail. Upon enrollment, oral mucosa examination revealed multiple white spots and a lower gingiva mass (**Figure 1B**). Tumor marker of CA125 and CA199 was slightly increased. Given her prior response to immunotherapy, we opted for intratumoral delivery of pembrolizumab (a PD1 inhibitor) plus ipilimumab every 3 weeks via oral tumor submucosal injection. After four treatments, her tumor marker CA-242 normalized from 41.0 to 16.1 U/mL (normal range: 0–29.0 U/mL), while CA199 decreased from 64.5 U/mL to 40.0 U/mL (normal range: < 35 U/mL) and remained slightly above the upper limit of normal, which may be associated with her history of hepatitis (HBV infection). The cheek and gingival lesions completely resolved (**Figure 1C**). As of the January 9, 2025, enhanced MRI showed no evidence of recurrence. Treatment-related adverse events (TRAEs) included grade 1–2 fever, elevated alkaline phosphatase, fatigue, hair loss, dry mouth, and puncture site swelling/pain. As of October 17, 2025, her OS and PFS were 65.70 months and 59.77 months, respectively.

**Figure 2:**
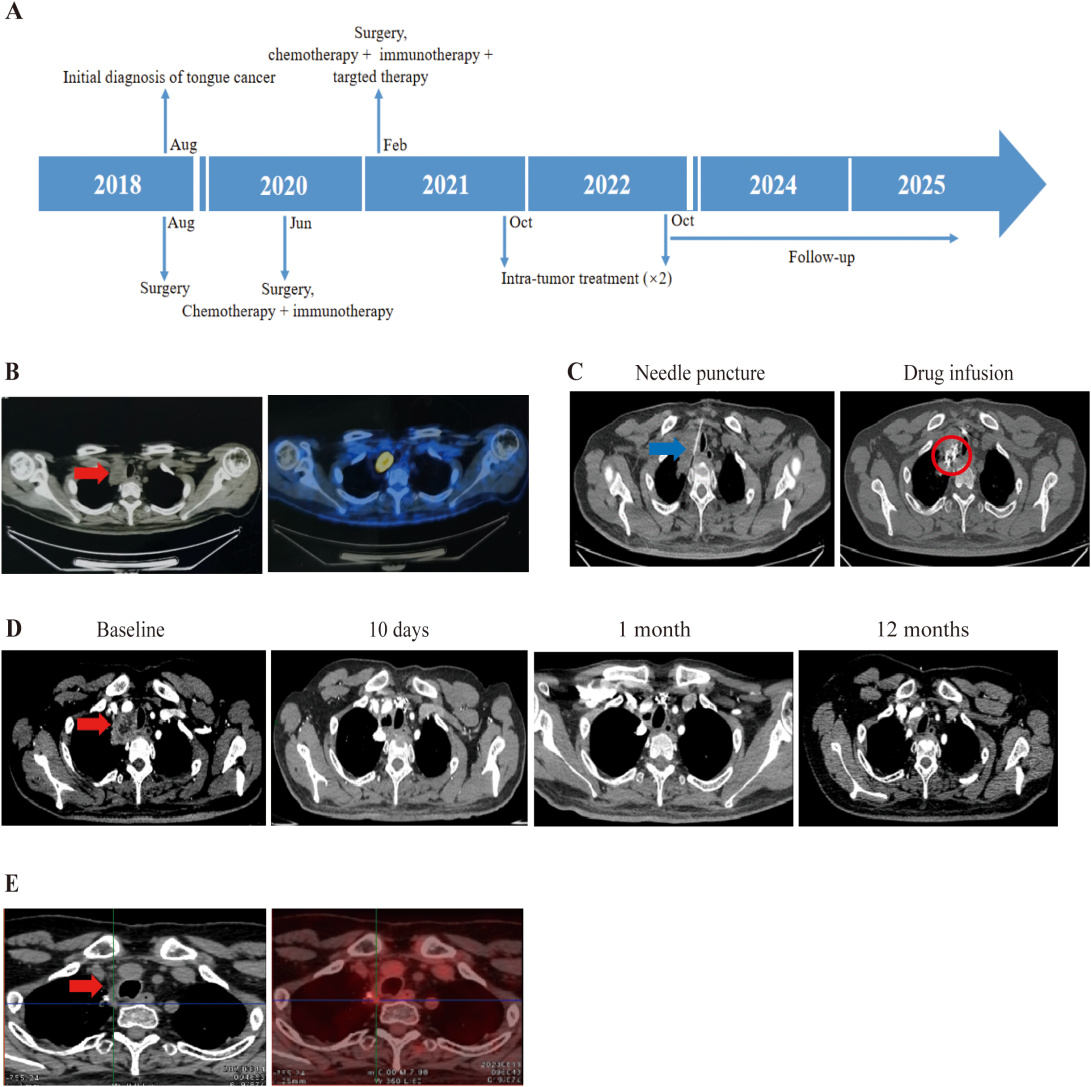
Treatment process of combination therapy for Patient 2. **(A)** Overview of the treatment timeline. **(B)** Prior to inclusion in clinical trials, PET-CT from external hospitals showed a metastasis between the right up lung and mediastinum with hypermetabolism. The tumor is indicated by the red arrow. **(C)** Intratumoral administration first involved using a Chiba needle to puncture the mediastinal tumor under CT guidance. An interventional radiologist determined the puncture point and path. In order to clarify the distribution of the injected drugs, contrast agent was added to the combination of 5ml durvalumab and 5ml ipilimumab, which presents as high density in CT. The needle is pointed out with blue arrow, and drug diffusion region is highlighted with red circle. **(D)** Enhanced CT image revealing mediastinal metastasis. Follow-up CT scans at 10 days, 1 month, and 12 months from baseline showed a disappearance of the lesion with no need to do further treatment. The tumor is indicated by the red arrow. **(E)** On August 11, 2023, PET-CT showed the tumor had disappeared with no significant activity.

Patient 2, a 39-year-old male was diagnosed with SCC of the right tongue in August 2018. He underwent primary tumor resection with skin flap repair and right neck lymph node dissection, with post resection pathological staging as pT3N0M0. In June 2020, a tumor recurred in his floor of the mouth and residual left tongue by MRI, followed by wide local excision (entire tongue, floor of mouth, partial mandible), bilateral neck lymph nodes dissection, and skin flap repair. Post surgical pathology confirmed recurrent SCC with nerve invasion, left neck level I/II lymph node metastasis, and negative surgical margins, pathologically staging as pT4N2M0. Post operation, he received four cycles of chemotherapy (albumin paclitaxel + nedaplatin) combined with nivolumab. In February 2021, right cervical lymph node metastasis was detected; biopsy-confirmed metastatic cancer prompted a third neck lymph node dissection. Then he completed four cycles of chemotherapy (cisplatin + albumin paclitaxel) combined with pembrolizumab and cetuximab (an EGFR monoclonal antibody) (**Figure 2A**). Despite treatment, disease progressed rapidly, with December 2021 PET-CT revealing an upper mediastinal metastatic lesion (**Figure 2B**). Given the ineffectiveness of his previous treatment, he enrolled in our trial on October 18, 2021. PD-L1 test in our center show TPS as 3%. Under CT guidance, durvalumab (a PD-L1 inhibitor) plus ipilimumab were injected into the mediastinal tumor through a fine needle (**Figure 2C**). 2 treatments were administered. Follow-up CT scans at 10 days, 1 month, and 12 months showed gradual lesion shrinkage (**Figure 2D**), and August 2023 PET-CT confirmed no residual tumor activity (**Figure 2E**). On March 19, 2025, an enhanced CT suggested tumor negative result. TRAEs included grade 3 lymphopenia and grade 1–2 leukopenia, hypothyroidism (according to CTCAE v5.0). As of October 17, 2025, his OS and PFS were 47.97 months and 27.23 months, respectively.

**Figure 3:**
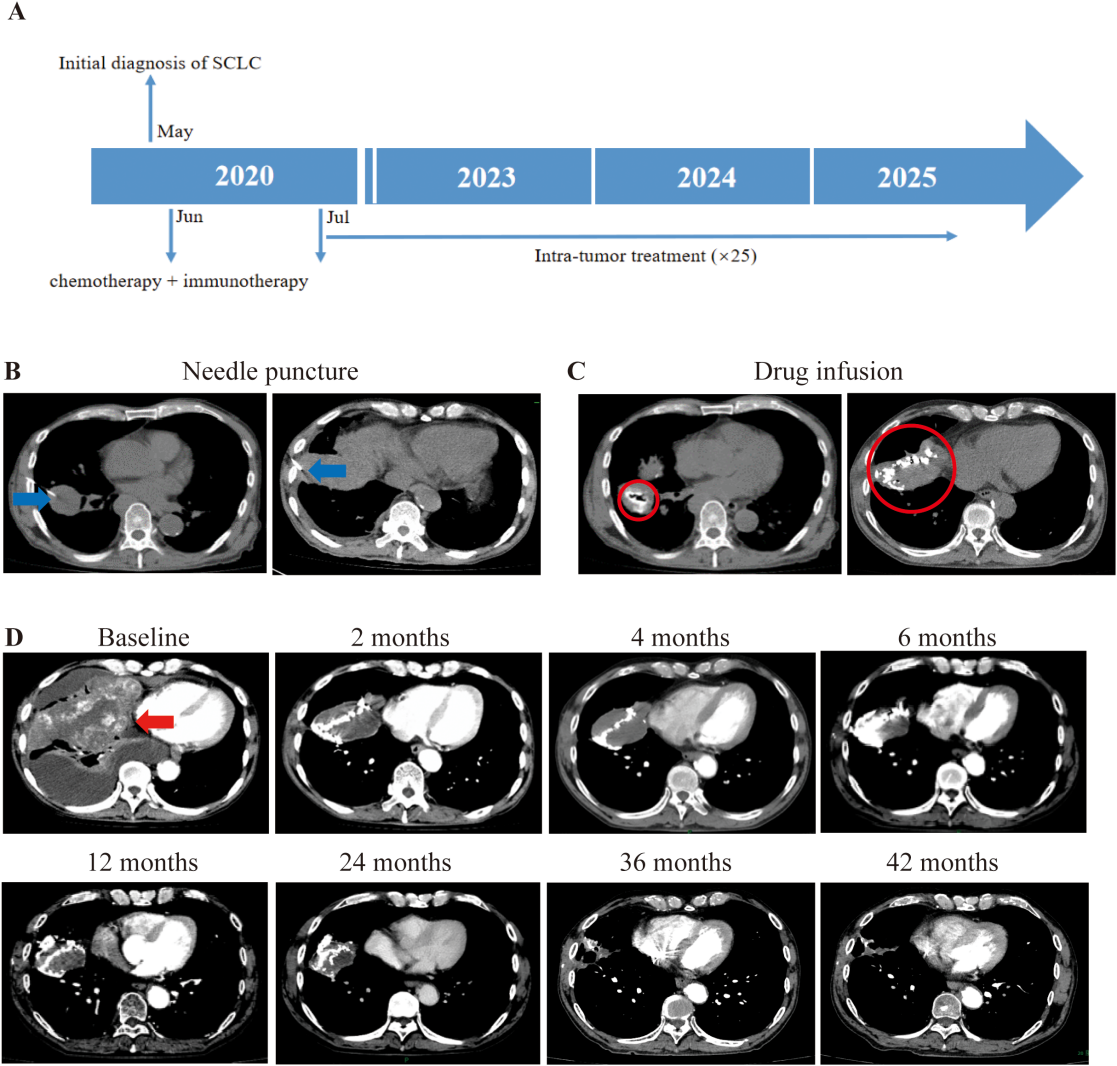
Illustration of the treatment process of combination therapy for Patient 3. **(A)** An overview of the treatment timeline is presented. **(B)** Based on the difficulty of puncture and the patient’s symptoms, we chose to inject the drug (5ml durvalumab+5ml ipilimumab) into the tumor in the right lung. The puncture needle is mainly selected based on the distance from the skin to the tumor. The needle is pointed out with a blue arrow. **(C)** The drug was injected into the interior of the cancer. To prevent drug leakage, CT scans are still required during the injection process. If there is a drug leak, surgery will be stopped depending on the situation. The drug diffusion region is circled by red circle. **(D)** Following treatment, the biggest tumor in the right lung gradually shrank over time, as indicated by the red arrow.

Patient 3, a 56-year-old male was diagnosed with right small cell lung cancer (SCLC) (cT4N2M1a) in May 2020. Initial antibiotic treatment for suspected severe infection was followed by chest CT, which revealed a huge right lung mass with right lung metastases, ipsilateral mediastinal and right hilum lymph node metastases, pericardial metastases, and malignant chest cavity effusion. In June 2020, bronchofiberoscopic biopsy confirmed right lung small cell carcinoma and PD-L1 expression was negative by IHC. He initiated chemotherapy (etoposide + lobaplatin + albumin paclitaxel) and immunotherapy (atezolizumab, a PD-L1 inhibitor) in June 2020 for one time and stopped due to poor physical condition and rapid tumor progression (**Figure 3A**). Despite the rapid progression of his disease, he maintained a strong desire for treatment and was therefore recruited into the study in July 2020. Upon enrollment in our trial, CT showed multiple right lung and mediastinal metastases, with the tumor occupying most of the right lung. On blood testing, the levels of CA125 and neuron-specific enolase (NSE) were found to be abnormal. From July 2020, intratumoral injection of half dose of durvalumab (a PD-L1 inhibitor) plus ipilimumab into the right lung primary tumor (and right chest cavity upon drainage of pleural effusion for first 3 times) (**Figures 3B, C**) had performed for 12 times with interval of 3–4 weeks until March 2021 while the primary tumor was visible and feasible for puncture. His right lung tumor gradually shrank until it disappeared. After that, maintenance immunotherapy was followed by intra-venous infusion of the same two drugs with the same half dose as previous intratumor injection, with interval of 2–3 months. In February 2023, CT revealed cavitary lung lesions, and sputum smear confirmed tuberculosis; anti-tuberculosis treatment was initiated upon the diagnosis of Tb. Due to sustained tumor shrinkage during 2-year follow-up, treatment intervals were extended to 3–4 months. At 42 months post-baseline, CT showed no visible tumor with only residual scars (**Figure 3D**). CA125 normalized from 156.8 to 5 U/ml (normal range: 0–35 u/ml). His NSE level returned to the normal range (decreased from 22.25 to 6.71 μg/L; normal range: < 15.2 μg/L) and remained within it for an extended period until he contracted tuberculosis. TRAEs included grade 1–2 hypotension, fever, thrombocytopenia, dizziness, fatigue, chills, pruritus, and hypothyroidism (according to CTCAE v5.0). As of October 17, 2025, his OS and PFS were 62.67 months and 32.13 months, respectively. Owing to concerns regarding disease progression, the patient insists on continuing the combination intravenous infusion of durvalumab and ipilimumab, with a treatment interval of 2–6 months.

**Figure 4:**
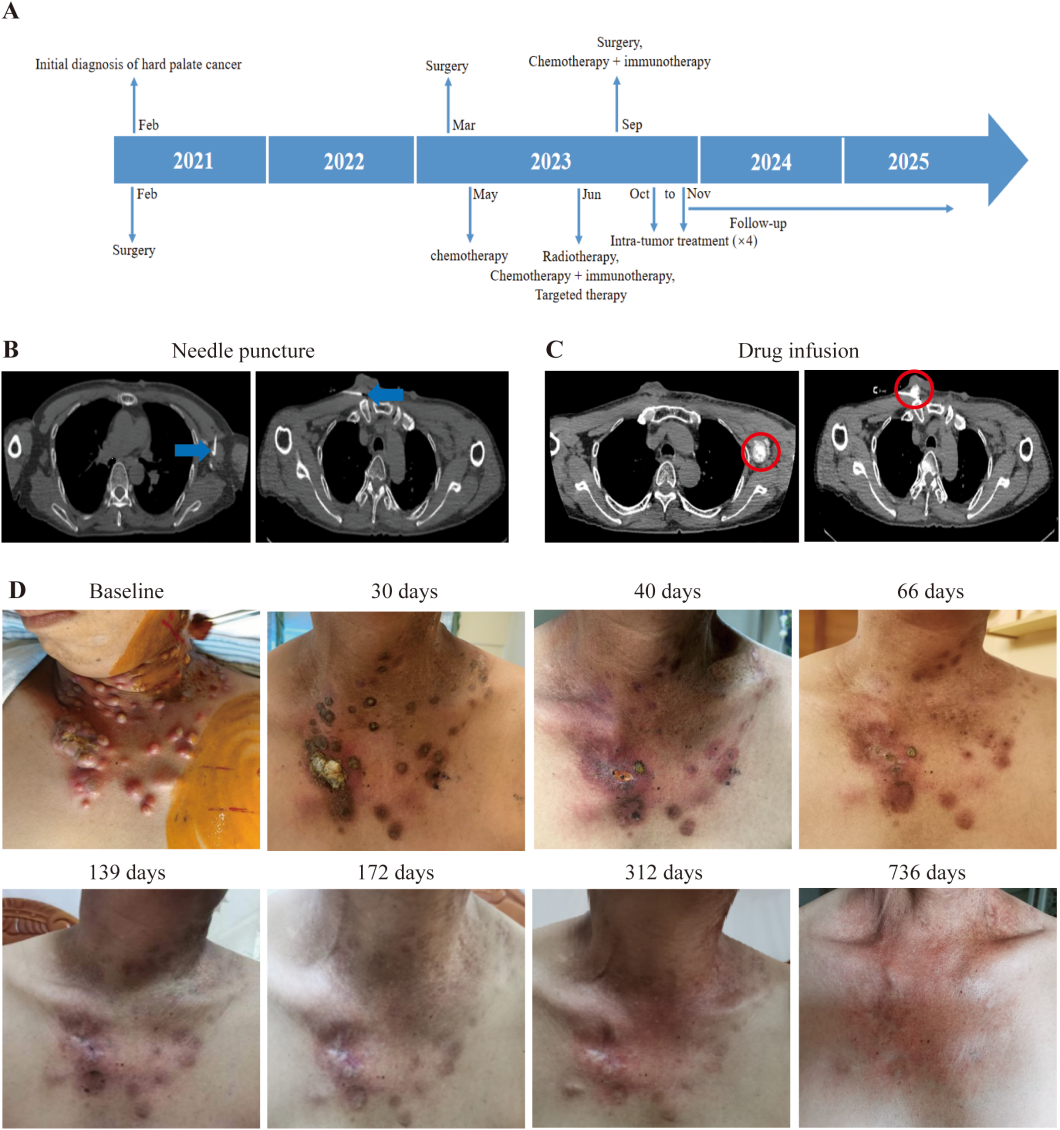
Illustration of the treatment process of combination therapy for Patient 4. **(A)** The treatment timeline of Patient 4 is presented. **(B)** For Patient 4, medication (5ml durvalumab + 5ml ipilimumab) was administered under CT guidance targeting the chest wall and axillary lymph nodes. The needle is pointed out with blue arrow. **(C)** After injecting the drug, a CT scan was performed to confirm the location of the drug. The drug diffusion region is circled by red circle. **(D)** Upon enrollment, multiple metastatic tumors were evident on the patient’s chest wall; however, with time, these tumors gradually diminished in size after the first treatment. The tumor site is beginning to be covered by newly formed skin.

Patient 4, a 52-year-old male diagnosed with SCC of the left oral hard palate in February 2021. He underwent a comprehensive treatment plan, encompassing removal of primary lesion, oral function reconstruction, and allograft dermal repair after primary diagnosis. Pathological examination of the primary lesion suggested well differentiated SCC with an invasion depth of 0.5cm, negative margins, and stage pT2N0M0. In May 2022, several nodules occurred in the left neck, but the CT scan at the local hospital suggested benign lesions. Until March 2023, the nodules greatly increased in size. MRI confirmed that these nodules were enlarged lymph nodes at the left neck regions II, III, and IV. So, he underwent a second surgery to dissection lymph nodes in the left neck. Pathology for lymph nodes is metastasis of moderately differentiated SCC. Adjuvant chemotherapy was initiated with a combination of cisplatin and paclitaxel in May 2023. On June 16, 2023, in addition to chemotherapy, the combination of camrelizumab (a PD-1 inhibitor) was administered. However, a PET-CT on June 20, 2023 revealed metastases in the left pharyngeal space, left neck, bilateral supraclavicular fossa, and left axilla. External beam radiation therapy (EBRT) was commenced on June 26, 2023 for the metastases on the left neck. In September 2023, a metastasis on his chest skin led to a local resection surgery, confirming metastatic SCC. Until September 2023, he had 30 times of EBRT and also received three injections of nimotuzumab (an EGFR inhibitor) with a frequency of once a month. On September 22, 2023, a combination therapy of “cisplatin + paclitaxel + camrelizumab” he had for one time (**Figure 4A**). All the aforementioned treatments proved ineffective, so he approached us and enjoined the trial. During the initial tumor assessment, his chest skin was covered with tumors (**Figure 4D**). We performed intratumoral delivery of dual immunotherapy (durvalumab + ipilimumab) with half dose of intra-venous injection four times into the biggest and feasible chest skin metastases and axillary lymph node metastases (**Figures 4B, C**). Due to the superficial and partially ruptured nature of the tumor on the chest wall, the drug may be more prone to leakage. Therefore, for chest wall tumors, only deeper tumors were injected. After the first treatment, all of the patient’s tumors started to shrink and gradually disappeared upon the time (**Figure 4D**). A PET-CT on September 2025 showed no sign of tumor in his whole body. As of 736 days (October 19, 2025), his thoracic skin recovered with normal appearance (**Figure 4D**). TRAEs include lymphopenia, decreased white blood cell count, pain at the puncture site, and fever, with lymphopenia graded 3 and the rest graded 1–2 (according to CTCAE v5.0). As of October 17, 2025, he achieved an OS of 24.16 months, PFS of 18.52 months.

## Discussion

Cancer remains a formidable challenge worldwide, particularly for patients with R/R advanced solid tumors, where effective treatment methods are still lacking. The emergence of PD-1/PD-L1/CTLA-4 inhibitors undoubtedly provide a new ray of hope for curing cancer, yet the response rate of patients remains disappointingly low (14). Clinical trials have shown that combination therapy with CTLA-4 and PD-1 has excellent therapeutic potential, which may be attributed to the complementary or even synergistic effects of each drug (15). However, intravenous injection leading significant side effects, and the penetration of drugs from the circulation into solid tumors are usually limited. The drug directly injected into the tumor will first spread to the entire injection area, where it reaches a very high initial tissue concentration locally, and then dissipates into the systemic circulation over time (16). In addition, for patients with polyclonal metastasis, multiple site injections may lead to a stronger adaptive immune response (17).

Here, we conducted a prospective clinical trial and administered PD-1/PD-L1 and CTLA-4 inhibitors via intratumoral delivery to patients with heavily treated advanced solid tumors. In our 4 cases reported here, they all achieved CR. Notably, patient 3 with extensive-stage small-cell lung cancer (ES-SCLC) attained an OS exceeding 5 years—remarkable given that the established 5-year survival rate for ES-SCLC is less than 7% (18). In previous studies, single immunization did not improve outcomes in locally advanced head and neck SCC (SCCHN) (19). Furthermore, the combination of durvalumab and tremelimumab failed to improve OS in patients with recurrent or metastatic SCCHN who had high PD-L1 expression (20). But in the three patients of SCCHN in our case achieved long-term remission. The concept of intratumoral injection has actually been proposed for a long time, but it has not been widely applied in clinical practice. There is currently no unified standard for the selection, puncture needle, drug dosage, etc. of injected tumors. There are also few studies reporting the clinical application of this technology. Intratumoral injection of ipilimumab was found in a study to increase the frequency of granzyme B- and/or perforin-positive CD8+ T cells in patients’ peripheral blood (21). We think repeated intratumoral injection of drugs may activates the patient’s specific anti-tumor immune system by turning cold tumors into hot ones. In subsequent research, it is imperative to gather blood and tissue samples from patients before and after treatment for the purpose of immunological analysis.

In addition, most TRAEs in our cases are of grade 1-2. Patients with grade 3 lymphopenia could be reversed during the interval after treatment, with no special treatment required. It must be emphasized here that after Patient 1 experienced a SAE, the patient was able to continue immunotherapy by changing the drug delivery approaches, which confirmed the advantage of intratumoral injection in reducing AEs. Common AEs reported with CTLA-4 inhibitors include colitis, hypophysitis, and rash, while pneumonia and hypothyroidism are common in patients using PD-1 inhibitors (22). However, no side effects attributable to the CTLA-4 antibody were observed. We believe that in addition to intratumoral injection, dosage is also one of the reasons for reducing AEs, as the incidence of CTLA-4 related AEs is directly proportional to dosage (23).

Of course, our research has indeed encountered some problems during its progression. For example, we have learned that half of the intravenous doses were safe for patients, but we did not consider tumor heterogeneity. How to select an appropriate injection site based on the patient’s condition; how to control drug dispersion and leakage; and which biomarkers can guide the selection of this tailored approach, all these remain unclear. This clinical trial is still under recruitment, and in the future we will further report complete data.

In summary, our research has confirmed the feasibility and potential clinical benefits of intratumoral injection, and provided theoretical basis for the development of this technology. This strategy may benefit individuals who have previously received intravenous ICIs but cannot tolerate adverse reactions, as well as those who are PD-L1 negative.

## Conclusions

Here, we have reported for the first time the data of intratumoral injection of PD-1/PD-L1 combined with CTLA-4 in patients with refractory advanced solid tumor. Our findings confirmed that intratumoral injection of ICIs is feasible in patients with advanced solid tumors. This technique may be more beneficial for activating tumor immunity and can effectively reduce drug-related AEs. Currently, large-scale clinical trials are underway, and we plan to report more accurate and authentic data in the future.

## Methods

### Study design

This research project is a single arm, single center, phase II clinical trial. The purpose is to evaluate the efficacy and safety of intratumoral injection of PD-1/PD-L1 combined with CTLA-4 inhibitors in the treatment of patients with advanced R/R solid tumors.

### Participants

A preliminary eligibility assessment will be conducted for each patient before study enrollment. Each patient will provide informed consent after receiving a thorough explanation of the study’s nature and before undergoing any study-specific procedures. Every patient will be informed about potential treatment side effects, the potential for unforeseen toxicities, and their right to discontinue participation or withdraw consent from the study at any time for any reason without prejudice toward further treatment.

The included patients are those over 18 years old who have been confirmed by histology or cytology to be unable to undergo surgical resection or have recurrent advanced solid tumors; At least one measurable lesion based on the RECIST 1.1; Acceptable heart, liver, and kidney function. Exclusion criteria include: 1. Uncontrolled infections, uncontrolled systemic diseases, or use of immunosuppressants or experimental drugs within the past 4 weeks; 2. Active autoimmune diseases, infections, cardiovascular diseases, or other serious acute or chronic illnesses; 3. Women who are pregnant, breastfeeding, or have tested positive for pregnancy; 4. Previous immune related toxicity ≥ grade 3-4 (according to CTCAE v5.0); 5. Patients receiving anticoagulant therapy or dual antiplatelet therapy that cannot be refused.

### Primary and second endpoints

The primary endpoint was progression-free survival (PFS). Secondary endpoints included duration of remission (DOR), overall survival (OS) and safety.

### Procedures

This treatment is administered every 3 weeks, and the duration can be appropriately shortened or extended depending on the patient’s tumor burden. To develop medication regimens and evaluate efficacy, patients need to undergo CT scans before each treatment. Before the initial treatment, PET-CT examination is required to understand the overall tumor situation. During the treatment period, all patients participating in this study will have their peripheral blood collected regularly (once every 3 weeks). The collected samples will be compared and analyzed with historical samples. This study used interventional methods through fine needle (22G-25G Chiba needles) to inject immune drugs into the tumor. The treatment plan was jointly determined by two oncology experts. The dosage of the drug is half of the intravenous injection dose specified in the drug instructions. The selection of immune drugs is mainly based on the patient’s previous use of immune drugs and PD-L1 expression. The volume of drug injected into a tumor is based on the diffusion of the drug within the tumor. After selecting the injection tumor, the drug will be injected into the tumor until the drug injection is complete or CT images show drug leakage.

In this study, security will be continuously monitored. Researchers assess the severity and relationship with the study treatment of AEs. According to CTCAE v5.0, adverse events will be classified into levels 0-5, with higher levels indicating more severe side effects. The tumor will be evaluated through CT scan according to RECIST v1.1 criteria. Researchers record the changes in the size of target and non-target lesions in patients over time. The patient undergoes tumor evaluation every 2 weeks starting from the first day of treatment, lasting for 48 weeks, and then every 4 weeks until PD, serious adverse events occur, or the patient withdraws consent.

### Statistical analysis

Descriptive statistics will be employed to summarize the clinical and biological characteristics.

Response and safety were evaluated according to RECIST v1.1 and CTCAE v5.0. According to RECIST v 1.1 criteria, CR: all target lesions have disappeared, and all pathological lymph nodes have been reduced to a normal size (short axis <10 mm); OS: the time from randomization to death or the last follow-up; PFS: The time between randomization and tumor progression, death, or the last disease assessment.

## Data Availability

The original contributions presented in the study are included in the article/supplementary material. Further inquiries can be directed to the corresponding authors.
